# Cancer Stemness Online: A Resource for Investigating Cancer Stemness and Associations with Immune Response

**DOI:** 10.1093/gpbjnl/qzae058

**Published:** 2024-08-14

**Authors:** Weiwei Zhou, Minghai Su, Tiantongfei Jiang, Yunjin Xie, Jingyi Shi, Yingying Ma, Kang Xu, Gang Xu, Yongsheng Li, Juan Xu

**Affiliations:** College of Bioinformatics Science and Technology, Harbin Medical University, Harbin 150081, China; College of Bioinformatics Science and Technology, Harbin Medical University, Harbin 150081, China; College of Bioinformatics Science and Technology, Harbin Medical University, Harbin 150081, China; College of Bioinformatics Science and Technology, Harbin Medical University, Harbin 150081, China; College of Bioinformatics Science and Technology, Harbin Medical University, Harbin 150081, China; College of Bioinformatics Science and Technology, Harbin Medical University, Harbin 150081, China; College of Bioinformatics Science and Technology, Harbin Medical University, Harbin 150081, China; College of Bioinformatics Science and Technology, Harbin Medical University, Harbin 150081, China; School of Interdisciplinary Medicine and Engineering, Harbin Medical University, Harbin 150081, China; College of Bioinformatics Science and Technology, Harbin Medical University, Harbin 150081, China

**Keywords:** Cancer stemness, Cancer stem cell, Single-cell RNA sequencing, Immunology

## Abstract

Cancer progression involves the gradual loss of a differentiated phenotype and the acquisition of progenitor and stem cell-like features, which are potential culprits of immunotherapy resistance. Although the state-of-the-art predictive computational methods have facilitated the prediction of cancer stemness, there remains a lack of efficient resources to accommodate various usage requirements. Here, we present the Cancer Stemness Online, an integrated resource for efficiently scoring cancer stemness potential at both bulk and single-cell levels. This resource integrates eight robust predictive algorithms as well as 27 signature gene sets associated with cancer stemness for predicting stemness scores. Downstream analyses were performed from five distinct aspects: identifying the signature genes of cancer stemness; exploring the associations with cancer hallmarks and cellular states; exploring the associations with immune response and the communications with immune cells; investigating the contributions to patient survival; and performing a robustness analysis of cancer stemness among different methods. Moreover, the pre-calculated cancer stemness atlas for more than 40 cancer types can be accessed by users. Both the tables and diverse visualizations of the analytical results are available for download. Together, Cancer Stemness Online is a powerful resource for scoring cancer stemness and expanding downstream functional interpretation, including immune response and cancer hallmarks. Cancer Stemness Online is freely accessible at http://bio-bigdata.hrbmu.edu.cn/CancerStemnessOnline.

## Introduction

Although numerous therapeutic modalities, such as surgery, radiation, chemotherapy and immunotherapy, have been developed to treat cancer, the risk of cancer recurrence remains high [1]. Cancer progression involves the gradual loss of a differentiated phenotype and the acquisition of progenitor and stem-cell-like features [2,3]. The existence of cancer stem cells (CSCs) has been reported in various cancer types [4]. Cancer stemness has also been reported to be a potential cause of immunotherapy resistance [5,6]. Therefore, a convenient platform that provides cancer stemness markers and a stemness index of patients or cancer cells is critical for understanding potential molecular mechanisms and developing effective therapies.

Recently, the state-of-the-art predictive computational methods have facilitated the assessment of cancer stemness. The majority of methods mainly rely on bulk or single-cell transcriptomes to evaluate the stemness of patients or cancer cells. Briefly, these methods can be classified into unsupervised and supervised methods. For example, the commonly used method is single-sample gene set enrichment analysis (ssGSEA) [7], which estimates the stemness score based on the expressions of collected stemness-related gene signatures. Moreover, cellular trajectory reconstruction analysis using gene counts and expression (CytoTRACE) has been recently developed to predict the differentiation and developmental potential of single cells by assessing the number of genes detected per cell [8]. Other tools, such as single cell lineage inference using cell expression similarity and entropy (SLICE) [9] and single-cell entropy (SCENT) [10], allow researchers to quantify stemness by entropy analysis. StemID [11] assesses the stemness of cell types within a population by utilizing tree topology and transcriptome composition.

On the other hand, numerous supervised methods have also been developed to estimate stemness. The mRNA stemness index (mRNAsi) is a widely used transcriptome stemness index to evaluate stemness based on the one-class logistic regression machine learning algorithm [12,13]. Relative expression orderings based on the stemness index (StemnessIndex) provide an absolute index to evaluate stemness by comparing the relative expression orderings of stem cell samples against normal adult samples from different tissues [13]. In addition, the stemness index for single-cell samples (StemSC) is a stemness index designed for single cells [14], which represents the percentage of gene pairs with the same relative expression orderings as the reference in embryonic stem cell samples. while these unsupervised and supervised methods provide valuable tools for estimating the stemness of patients or single cells, they are scattered across different studies and can be challenging for researchers without programming experience to use.

Some web servers or databases have been developed to depict cell stemness or collect stem cell-related data. However, the majority of these resources focus only on stem gene sets, and do not provide the stemness of samples from public data directly. For example, SISTEMA [15] collects a large number of human stem cell transcriptome data to display the expression of stem genes in different cell lines, cell types, and pathological conditions. StemMapper [16] collects transcriptome datasets of various stem cells. However, there is currently no efficient database that can meet various requirements of users.

Therefore, we developed Cancer Stemness Online (http://bio-bigdata.hrbmu.edu.cn/CancerStemnessOnline), which is a resource providing cancer stemness score (CSscore), functional analysis, and visualization. To assess the CSscore for bulk RNA sequencing (RNA-seq) or single-cell RNA sequencing (scRNA-seq) data, Cancer Stemness Online integrates five unsupervised methods and three supervised methods, which evaluate the differentiation level on the basis of transcriptional complexity or similarity to the reference profiles of stem cells. Basic statistical analysis and additional five advanced analysis modules are provided. Cancer Stemness Online is accessible online without registration and allows users to upload their data for analysis. Cancer Stemness Online provides multiple visualizations of the results for a better understanding of stemness. All charts and tables are available for download. Together, Cancer Stemness Online is a powerful resource for estimating cancer stemness and facilitates in-depth and broad downstream functional interpretation, including immune response and cancer hallmarks.

## Method

### Collection of cancer stemness gene sets

To collect the cancer stemness-related gene sets, we searched PubMed on December 10, 2019 for studies published since January 1, 2003 with “cancer stem cell” or “stemness” as keywords. The search was repeated on December 26, 2023 for update. In total, we manually curated 2860 articles and recorded 27 canonical cancer stemness gene sets (Table S1). The number of genes in these gene sets ranged from 5 to 1007. All gene names were mapped to classical gene symbols.

### Quality control

For the scRNA-seq data, we removed cells with less than 200 total counts and genes expressed in less than 3 cells. Cells with more than 5% mitochondrial gene counts were filtered out. For the bulk RNA-seq data, samples with no expressed genes were removed. To address the effects of noise and batching of the data, users can use several available tools, such as Seurat [17] and Harmony [18], before uploading it to Cancer Stemness Online.

### Calculation of CSscore

Cancer Stemness Online collected eight computational methods to evaluate the stemness potential based on multiple principles. These methods are categorized into unsupervised and supervised methods according to the reference of CSCs. On the other hand, mRNAsi [12], StemnessIndex [13], and gene set variation analysis (GSVA) [19] were applied to bulk RNA-seq data; CytoTRACE [20], SLICE [9], SCENT [10], StemSC [14], and GSVA [19] were used for the scRNA-seq data. To improve the comparability of results, we performed zero-one normalization to all CSscores. In addition, the CSscores of scRNA-seq data uploaded by users can also be calculated based on StemID [11].

### Single-cell trajectory analysis

To analyze the cell pseudotime in the scRNA-seq data, we performed “Monocle 2” [21], which uses reversed graph embedding to describe multiple fate decisions in a fully unsupervised manner.

### Identification of the stemness-related signatures

To assess the relevance between CSscores and gene expression, we calculated the Spearman correlation coefficient (SCC). The genes with false discovery rate (FDR) < 0.05 and SCC > 0.5 (default) were identified as cancer stemness-related gene signatures.

### Functional correlations

To investigate the functions of cell types, we first calculated the ssGSEA score for each cell [19]. The cellular states, immune signatures, and cancer hallmarks were considered. In addition, we calculated the SCC between the CSscores and ssGSEA scores. For bulk data, we calculated the infiltration of immune cells in the sample by “cell-type identification by estimating relative subsets of RNA transcripts (CIBERSORT)” function [22]. The SCCs between the CSscores and infiltrations of various immune cells were calculated.

### Robust rank aggregation

To comprehensively evaluate the CSscores of samples or cells derived from multiple computational stemness assessment methods, we employed Robust Rank Aggregation (RRA) [23] to integrate the ranking results. This integration provides an overall stemness score for each sample or cell. We subsequently calculated the SCCs between the integrated scores and individual CSscores from various computational methods.

### Survival analysis

The clinical information including overall survival and the state of samples can be uploaded by users. We applied the Cox proportional hazards regression model to assess the prognosis of all cancer stemness genes, based on their median expression. The Kaplan–Meier survival curves were generated by the “survminer” function with corresponding log-rank *P* values. For the genes with a positive beta value of “coxph”, we defined them as risk factors, and the negative ones were protective factors.

### Cell–cell communication

To further explore the interactions between CSCs and other cells, we identified cell–cell communications by identifying and illustrating alterations in the intercellular signaling network (iTALK; https://github.com/Coolgenome/iTALK). The integrated ligand–receptor interaction data were obtained from CellChat [24], CellTalkDB [25], ICELLNET [26], iTALK, NicheNet [27], SingleCellSignalR [28], and one recent study [29]. The union sets of ligand–receptor pairs were integrated into Cancer Stemness Online.

### Database implementation

The frontend of Cancer Stemness Online was built with hypertext markup language 5 (HTML5), JavaScript (JS), and cascading style sheets (CSS), and it included the jQuery (v3.3.1), DataTables (1.10.25), ECharts (v5.5.1), and D3 (v7.6.1) plugins. The backend was powered by Eclipse (Mars.2) and was queried via Java Server Pages with the Apache Tomcat container (v6.0) as the middleware. All data in Cancer Stemness Online were stored and managed using Eclipse (Mars.2), which employs Java and R programs to perform online analyses. Cancer Stemness Online has been tested on several popular web browsers, including Google Chrome, Mozilla Firefox, and Apple Safari.

## Results

### Overall architecture of Cancer Stemness Online

**Figure 1:**
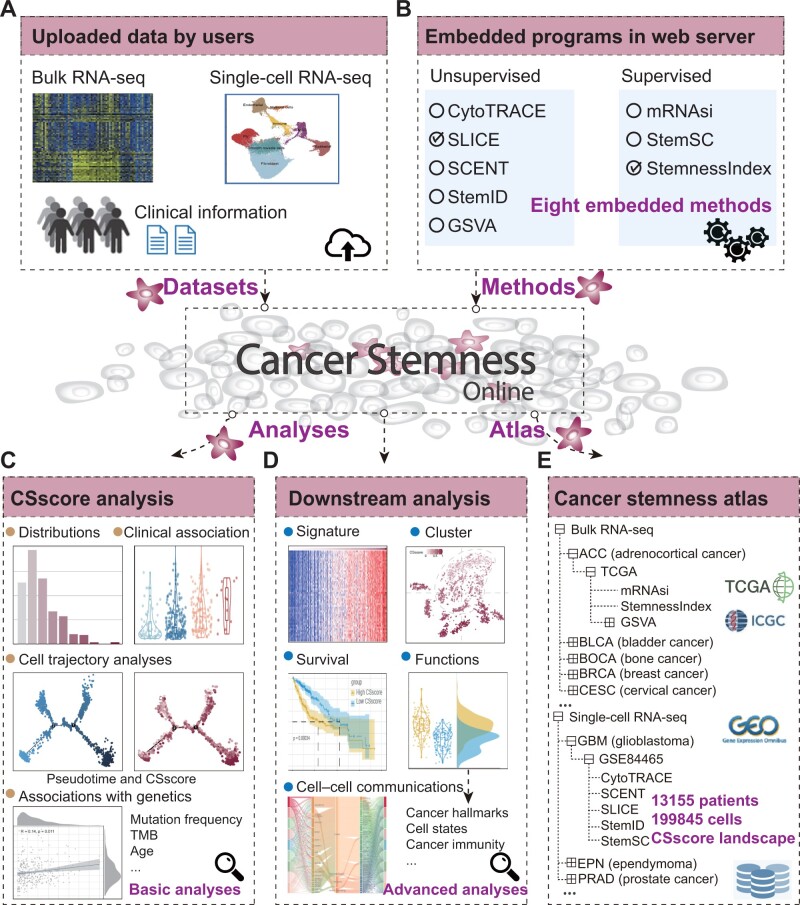
Overall architecture of Cancer Stemness Online **A**. Datasets uploaded by users, including transcriptomes or clinical information. **B**. Robust computational methods embedded in the platform. **C**. Basic analysis of cancer stemness in the database. **D**. Advanced downstream analysis of the stemness and its associations with clinical and genetic features. **E**. The cancer stemness atlas provided in Cancer Stemness Online. RNA-seq, RNA sequencing; CSscore, cancer stemness score; TMB, tumor mutational burden; TCGA, The Cancer Genome Atlas; GEO, Gene Expression Omnibus; ICGC, International Cancer Genome Consortium; CytoTRACE, cellular trajectory reconstruction analysis using gene counts and expression; SLICE, single cell lineage inference using cell expression similarity and entropy; StemnessIndex, relative expression orderings based on stemness index; StemSC, stemness index for single-cell samples; mRNAsi, mRNA stemness index; GSVA, gene set variation analysis; SCENT, single-cell entropy.

Cancer Stemness Online is designed to facilitate the prediction of the CSscore of tumor cells or samples. The overall design of this platform is summarized in **Figure 1**. This platform accommodates different types of transcriptomes uploaded by users, such as bulk RNA-seq and scRNA-seq data (Figure 1A). In addition, the users can also upload the patients’ clinical data. The input files can be prepared following the format description.

The platform integrates eight robust computational algorithms to predict the CSscore for each patient or cell. These methods are classified into five unsupervised methods and three supervised methods (Figure 1B). To facilitate the selection of methods, we provided practical guidance from two aspects: “By Model Type” and “By Input Type”. Next, the server executes the prediction of CSscore with the selected method. The distributions of CSscores, clinical associations, cell trajectory, and associations with genetic features are presented on the results page (Figure 1C). Furthermore, the downstream module can identify the gene signatures associated with CSscores, cluster the cells based on expression of gene signatures, perform survival analysis and functional prediction, and identify cell–cell communications (Figure 1D).

In addition to the interactive web interface, Cancer Stemness Online provides flexible ways to access the annotation of CSscore for available cancer transcriptome projects (Figure 1E), such as The Cancer Genome Atlas (TCGA), International Cancer Genome Consortium (ICGC), and single-cell transcriptomes from published studies. All the analysis results and visualization modules from the resource can be exported as high-quality images and downloaded for further analysis.

### User interface of Cancer Stemness Online

**Figure 2:**
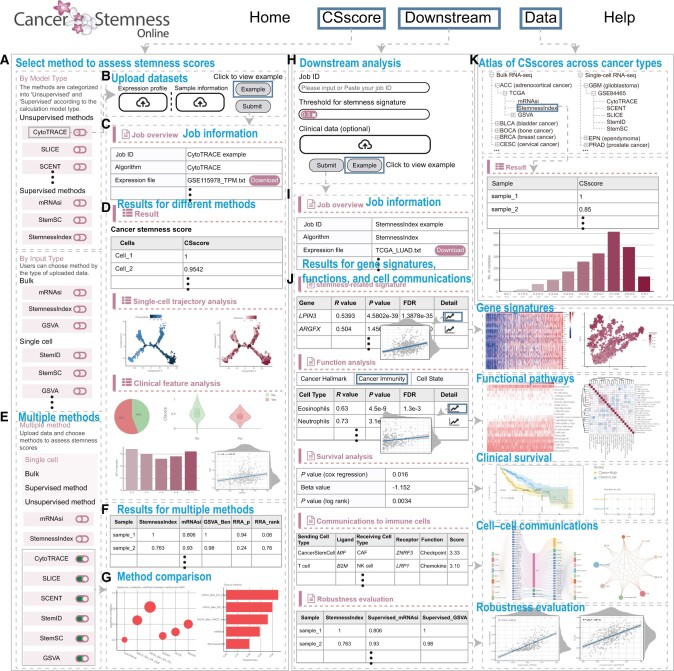
Interactive web interface of Cancer Stemness Online **A**. The methods provided in the platform for users, including unsupervised methods and supervised methods. **B**. The data upload page of the platform. **C**. Information on the user-submitted job. **D**. Results of the basic analysis of CSscores for the bulk and single-cell transcriptomes. **E**. Screenshots of the selection page for multiple methods. **F**. Results for multiple methods. **G**. Method comparison for different methods. **H**. Job submission page for the “Downstream” module. **I**. Detailed information for the user-submitted job. **J**. Results of advanced analysis, including heatmap of gene signatures, functional pathways and immune regulation, clinical survival, cell–cell communications, and robustness evaluation. **K**. CSscores across different cancer types provided in the platform.

Cancer Stemness Online is an open-access online platform for predicting the CSscore for cancer patients or cells. The web interface is freely available, and no login is required. The main features of Cancer Stemness Online are the “CSscore” and “Downstream” modules (**Figure 2**). Users can start predicting the CSscore by clicking the “GET STARTED” button on the homepage or by navigating directly to the “CSscore” module. The server allows users to predict the CSscore by selecting from the model type or input type (Figure 2A). In the “By Model Type” module, five unsupervised methods and three supervised methods can be selected. In the “By Input Type” module, three methods are suitable for bulk transcriptomes, and six methods are suitable for single-cell transcriptomes. The transcriptomes and clinical information of samples can be uploaded, and the users can also leave email information to further retrieve the results from email (Figure 2B).

The results page first returns the job information, such as the job ID, algorithm, and expression profiles (Figure 2C). The predicted CSscores and their associations with clinical features (*i.e.*, grade, tumor mutation burden, and treatment) are provided and visualized in the database (Figure 2D). We also provide a “Multiple method” module on the “CSscore” page, which allows users to select multiple methods and obtain the integrated rank of samples or cells based on the RRA algorithm (Figure 2E–G). Moreover, users can perform further downstream analyses from the “Downstream” module. Users can retrieve the predicted CSscores by inputting the job ID (Figure 2H). Several parameters can be selected, and additional clinical data can be optionally uploaded. New job information will first be provided (Figure 2I), and advanced analysis results will be provided in tables or images (Figure 2J). For example, genes associated with CSscores are provided in a table, and gene expression levels are visualized by a heatmap. The functional pathways enriched by gene signatures are also provided in table and heatmap formats. The clinical survival analysis was performed to evaluate the correlations between CSscores and patient survival (Figure 2J). Cell–cell communications and the correlations of CSscores predicted by different methods are also analyzed automatically in Cancer Stemness Online. In addition, the predicted CSscores of the TCGA, ICGC, and single-cell transcriptomes from published studies can be accessed from the “Data” module (Figure 2K). Users can find additional information from the “Help” page.

### Case study 1: cancer stemness analysis of bulk transcriptomes

**Figure 3:**
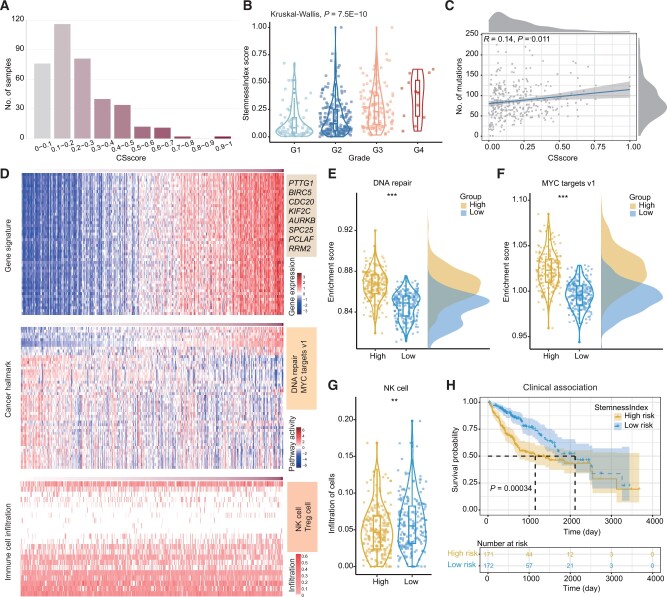
Cancer stemness analysis of bulk transcriptomes in HCC **A**. Distribution of CSscores across HCC patients. **B**. StemnessIndex scores of patients in different grades. **C**. Scatter plot showing the Spearman correlation between StemnessIndex scores and the number of mutations in cancer patients. **D**. Heatmaps showing the expression of gene signatures, activities of cancer hallmark pathways, and infiltration of immune cells. **E**. Box plot showing the enrichment scores of the DNA repair pathway in patients with high or low StemnessIndex scores. **F**. Box plot showing the enrichment scores of the MYC targets v1 pathway in patients with high or low StemnessIndex scores. **G**. Box plot showing the infiltration of NK cells in patients with high or low StemnessIndex scores. **H**. Kaplan–Meier curve for overall survival of patients with high or low StemnessIndex scores. HCC, hepatocellular carcinoma; NK, natural killer.

To illustrate the various functions of Cancer Stemness Online, we first analyzed the bulk transcriptomes of hepatocellular carcinoma (HCC) samples from TCGA [30]. We predicted the CSscore for each patient based on the StemnessIndex algorithm (**Figure 3**). The results showed that the majority of the patients had low CSscores (Figure 3A), although several patients exhibited high cancer stemness. The server also evaluated the associations between CSscores and clinical features. Cancer patients in high grade had significantly higher CSscores in HCC (Figure 3B). The CSscores of cancer patients were positively correlated with the number of mutations (*R* = 0.14, *P* = 0.011; Figure 3C), which is consistent with previous studies [12,13,31]. These results suggest that the CSscore is associated with clinical and genetic features in HCC.

Next, we performed advanced analyses based on the “Downstream” module of Cancer Stemness Online. We identified numerous genes whose expression levels were associated with CSscores in HCC (Figure 3D), including *BIRC5* [32], *CDC20* [33], *PTTG1* [34], and *KIF2C* [35]. Functional analyses revealed that the “DNA repair” and “MYC targets v1” pathways as well as the infiltrations of several immune cells were significantly associated with CSscores of cancer patients (Figure 3D). In particular, cancer patients with high CSscores exhibited significantly higher enrichment scores of “DNA repair” (Figure 3E; *P* < 0.001) and “MYC targets v1” (Figure 3F; *P* < 0.001). In addition, cancer patients with low CSscores displayed significantly greater infiltration of natural killer (NK) cells (Figure 3G; *P* < 0.01). Finally, we evaluated the survival rates of patients with different CSscores and found that patients with higher stemness exhibited significantly poorer survival in HCC (*P* = 0.00034, log-rank test; Figure 3H). These results suggest that Cancer Stemness Online not only accurately predicts cancer stemness but also provides novel insights into the functional pathways and immune regulation associated with cancer.

### Case study 2: cancer stemness analysis of single-cell transcriptomes

**Figure 4:**
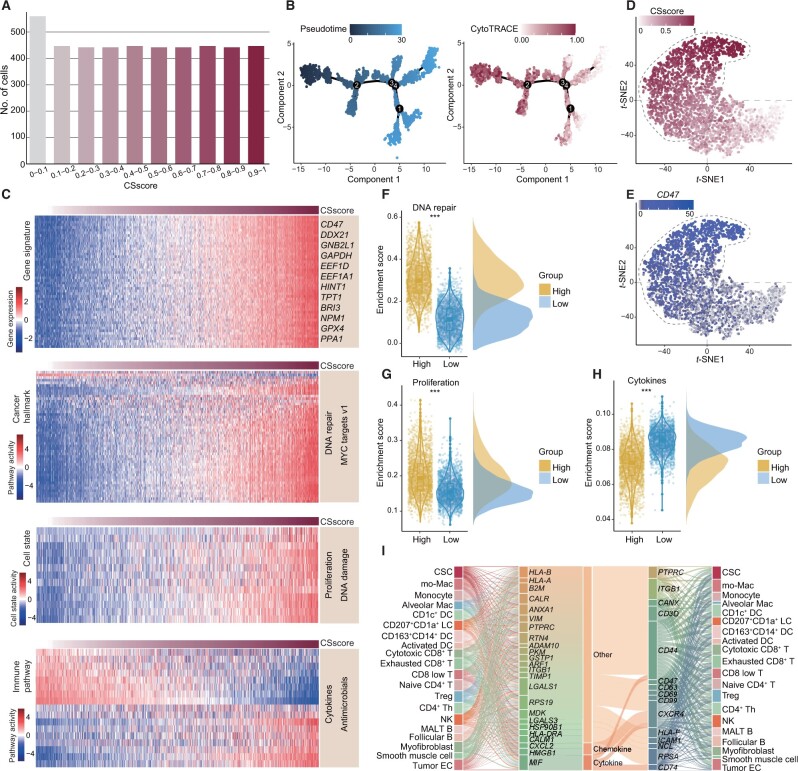
Cancer stemness analysis of single-cell transcriptomes in LUAD **A**. Number of cells with different CSscores. **B**. Lineage trajectory showing cells with different pseudotime and CytoTRACE scores. **C**. Heatmaps showing the expression of gene signatures, activities of cancer hallmark pathways, activities of cell states, and, activities of immune pathways. **D**. *t*-SNE plot showing the distribution of cells based on the expression levels of gene signatures. **E**. *t*-SNE plot showing the distribution of cells based on the expression of *CD47*. **F**. Box plot showing the enrichment scores of the DNA repair pathway in cancer cells with high or low CSscores. **G**. Box plot showing the enrichment scores of proliferation in cancer cells with high or low CSscores. **H**. Box plot showing the enrichment scores of the cytokines pathway in cancer cells with high or low CSscores. **I**. Cell–cell communications mediated by ligand–receptor interactions. LUAD, lung adenocarcinoma; *t*-SNE, *t*-distributed stochastic neighbor embedding; CSC, cancer stem cell; mo-Mac, monocyte-derived macrophage; Mac, macrophage; DC, dendritic cell; LC, Langerhans cell; Treg, regulatory T cell; Th, helper T cell; EC, endothelial cell.

The development of single-cell sequencing in cancer research has revolutionized our understanding of the biological characteristics of different cancer types [36]. We next analyzed the cancer stemness of single-cell transcriptomes based on the Cancer Stemness Online server. We obtained the single-cell transcriptomes of lung adenocarcinoma (LUAD) and melanoma samples from previous studies [23,37]. The CSscore for each cancer cell was estimated based on the CytoTRACE algorithm embedded in the server (**Figure 4**, Figure S1). The results showed that large numbers of cells had higher CSscores in LUAD and melanoma (Figure 4A, Figure S1A). In addition, the pseudotime of cells was estimated by Monocle 2, and we found that cells with low pseudotime presented significantly higher CSscores (Figure 4B, Figure S1B). Immune checkpoint inhibitors (ICI) produce durable responses in some melanoma patients. We observed that cells from post-treatment exhibited significantly higher CSscores than those of treatment-naive (*P* < 2.2E−16; Figure S1C), suggesting potential immunotherapy resistance [31].

In the “Downstream” module, we identified numerous genes whose expression levels were correlated with CSscores (Figure 4C, Figure S1D). We found that the expression of genes can effectively distinguish cells with higher or lower CSscores (Figure 4D, Figure S1E). For example, *CD47* (Figure 4E) and *BIRC5* (Figure S1F) were highly expressed in cells with higher CSscores, confirming previous findings [23]. Functional pathway and immune regulation analyses revealed that cancer cells with high CSscores were significantly enriched in the DNA repair pathway (*P* < 0.001; Figure 4F, Figure S1G) and proliferation (*P* < 0.001; Figure 4G, Figure S1H), while the cytokines pathway exhibited the opposite pattern (*P* < 0.001; Figure 4H). These results are consistent with previous observations [31,38,39]. We further investigated the cell–cell communications based on the ligand–receptor interactions, and found that CSCs communicated with other immune cells via various ligand–receptor interactions (Figure 4I, Figure S1I). In particular, the interaction between ADAM10 and CD44 facilitated the communication between CSCs and T cells (Figure 4I, Figure S1I) [40,41]. All the analysis results visualized on the web interface are available for download.

## Discussion

Cancer Stemness Online is a useful resource for scoring cancer stemness and its associations with the immune response, and integrates eight robust predictive algorithms. The platform supports different types of input transcriptomes, and the output of Cancer Stemness Online provides tables and images for visualization of the CSscores and their associations with clinical features. These results can help noncomputational biologists explore cancer stemness effectively. In addition, Cancer Stemness Online not only encompasses diverse functionalities but also offers user-friendly operations and visually intuitive interfaces. Recent studies have shown that a high stemness profile in cancer is associated with an inferior immunogenic response [42]. Different types of immune cells can be recruited from tumor-associated stem cells [43–45]. Thus, the “Downstream” module in Cancer Stemness Online provides advanced analysis for investigating functional pathways and immune regulation in the context of cancer stemness. Overall, Cancer Stemness Online is a user-friendly platform for predicting cancer stemness and exploring its functional consequences in cancer.

We provide diverse methods to predict the stemness scores of individual samples or cells. To assist the users in selecting appropriate methods, we compared the performances of different algorithms based on both bulk and single-cell transcriptomes from recent studies [46,47]. Our findings suggest that the StemnessIndex method may be the most effective method for bulk transcriptomes, whereas CytoTRACE appears the most effective method for single-cell transcriptomes (Figure S2). In addition, we provide a “Multiple method” module on the “CSscore” page. This module allows users to select multiple methods to predict stemness scores and obtain the integrated rank of samples or cells based on the RRA algorithm. The runtime and correlations between different methods and RRA are also provided. Thus, users can integrate the results from multiple methods for downstream analysis.

Nevertheless, there is still room for improvement in the future. Here are a few areas that we plan to expand in the future version of Cancer Stemness Online. (1) Improving the coverage of computational methods and cancer stemness gene sets. Currently, eight computational methods are integrated, and we aim to cover newly developed algorithms and cancer stemness gene sets in the near future. (2) Expanding to cover additional genomes and transcriptomes. The server currently only predicts the CSscores for human transcriptomes, which should be considered in the future working for a wider range of species. With the development of high-throughput sequencing technology, additional cancer transcriptomes will be added to the cancer stemness atlas. (3) Including additional annotations. We plan to add more functional annotations, such as more immune cells, pathways, and immunotherapy-related information.

Overall, Cancer Stemness Online is a powerful resource for reducing the barrier to analyze the enormous amount of transcriptome data that biomedical researchers face and facilitating the identification of associations with cancer immunotherapy response for further mechanistic and functional insights.

## Supplementary Material

qzae058_Supplementary_Data

## Data Availability

The web server of Cancer Stemness Online is freely accessible at http://bio-bigdata.hrbmu.edu.cn/CancerStemnessOnline.

## Code availability

The code used to perform the analyses in this study is available at https://github.com/ComputationalEpigeneticsLab/CancerStemnessOnline.

## CRediT author statement

**Weiwei Zhou:** Conceptualization, Software, Formal analysis, Visualization, Methodology, Writing – original draft. **Minghai Su:** Conceptualization, Software, Formal analysis, Visualization, Methodology, Writing – original draft. **Tiantongfei Jiang:** Conceptualization, Software, Formal analysis, Visualization, Methodology, Writing – original draft. **Yunjin Xie:** Conceptualization, Methodology, Writing – review & editing. **Jingyi Shi:** Software, Methodology, Writing – review & editing. **Yingying Ma:** Conceptualization, Software, Methodology, Writing – review & editing. **Kang Xu:** Conceptualization, Software, Methodology, Writing – review & editing. **Gang Xu:** Conceptualization, Software, Writing – review & editing. **Yongsheng Li:** Conceptualization, Formal analysis, Supervision, Methodology, Project administration, Writing – review & editing. **Juan Xu:** Conceptualization, Formal analysis, Supervision, Methodology, Project administration, Writing – review & editing. All authors have read and approved the final manuscript.
