# Supplementary material for: Cancer Stemness Online: A Resource for Investigating Cancer Stemness and Associations with Immune Response
Source: Genomics Proteomics Bioinformatics. 2024 Aug 14;22(4):qzae058. doi: 10.1093/gpbjnl/qzae058 (PMC11522875; doi:10.1093/gpbjnl/qzae058)
Supplement: qzae058_Supplementary_Data [file qzae058_supplementary_data.zip › supplementary material captions.docx]

**Supplementary material**

**Figure S1 Cancer stemness analysis of single-cell transcriptomes in melanoma**

**A.** Number of cells with different CSscores. **B.** *t*-SNE plot showing the cells with different pseudotime and CytoTRACE scores. **C.** Distribution of CSscores for cells from post treatment and naive. **D.** Heatmap showing the expressions of gene signatures, activities of cancer hallmark pathways or cell states, and immune pathways. **E.** *t*-SNE plot showing the distribution of cells based on expressions of gene signatures. **F.** *t*-SNE plot showing the distribution of cells according to the expression of *BIRC5*. **G.** Box plots showing the enrichment scores of the DNA repair pathway in cancer cells with high or low CSscores. **H.** Box plots showing the enrichment scores of proliferation in cancer cells with high or low CSscores. **I.** Cell–cell communications mediated by ligand–receptor interactions. CAF, cancer-associated fibroblasts; NK, natural killer cells.

**Figure S2 Accuracy of cancer stemness methods**

**A.** The correlation between CSscores and differentiation days as calculated by the three bulk methods. **B.** The correlation between CSscores and differentiation days as calculated by the six single cell methods. The score was calculated using Spearman’s correlation coefficient.

**Table S1 Stemness marker gene sets used in this study**
