# Supplementary figures and images for: Cancer Stemness Online: A Resource for Investigating Cancer Stemness and Associations with Immune Response
Source: Genomics Proteomics Bioinformatics. 2024 Aug 14;22(4):qzae058. doi: 10.1093/gpbjnl/qzae058 (PMC11522875; doi:10.1093/gpbjnl/qzae058)

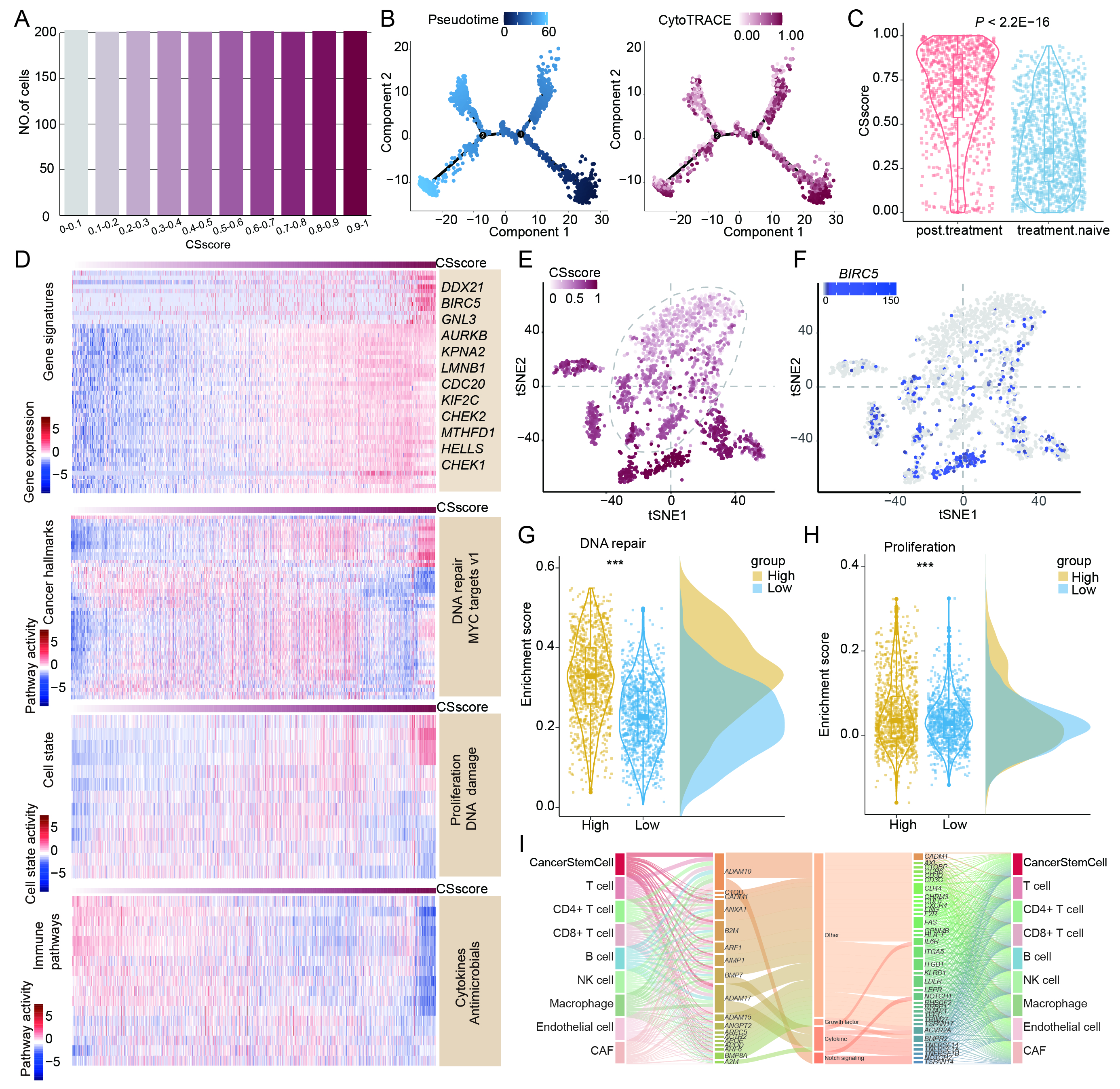

Supplement: qzae058_Supplementary_Data [file qzae058_supplementary_data.zip › Figure-S1.jpg]

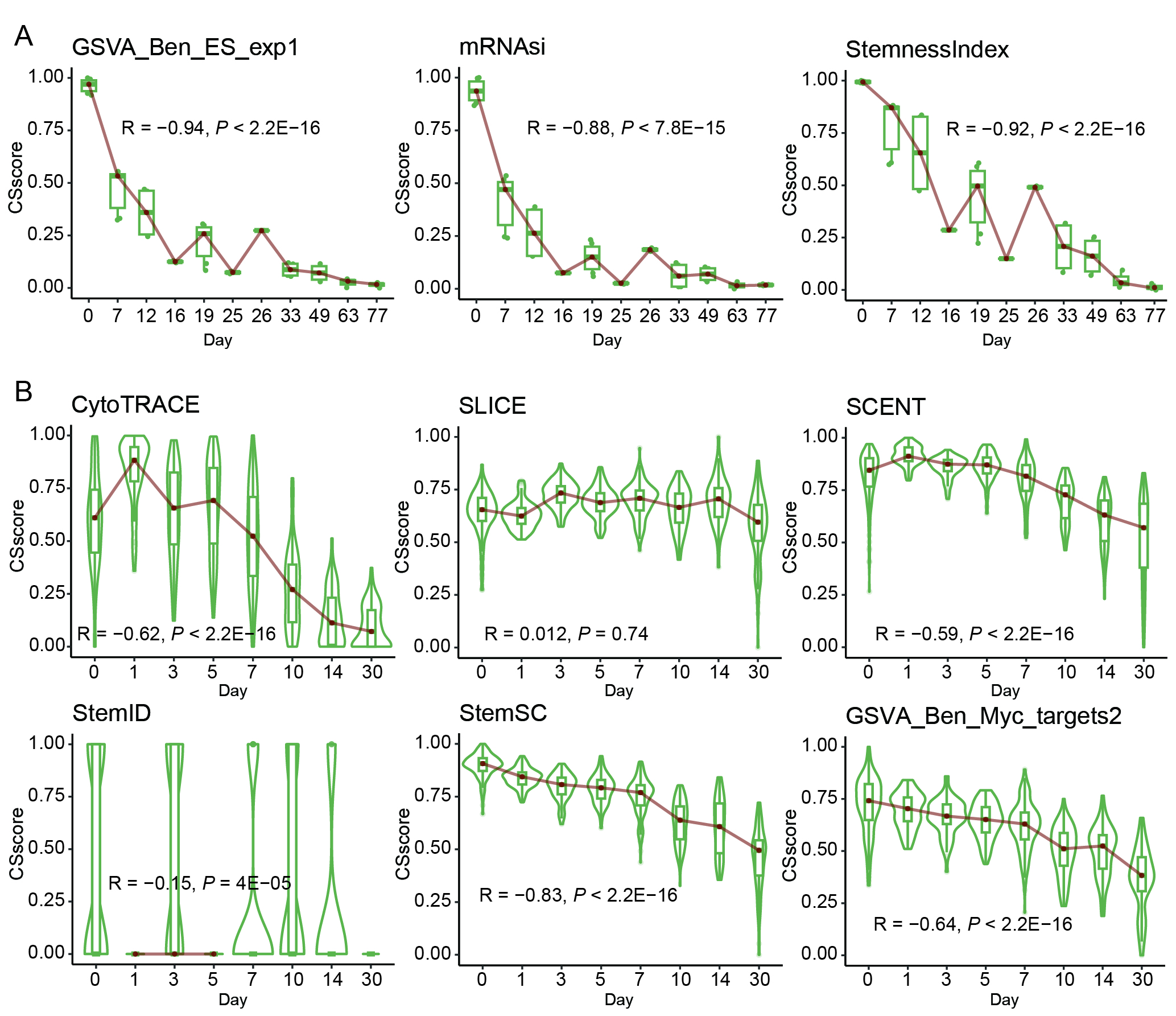

Supplement: qzae058_Supplementary_Data [file qzae058_supplementary_data.zip › Figure-S2.jpg]
